# Non-invasive monitoring of T cell differentiation through Raman spectroscopy

**DOI:** 10.1038/s41598-023-29259-8

**Published:** 2023-02-22

**Authors:** Nicolas Pavillon, Nicholas I. Smith

**Affiliations:** 1 Biophotonics Laboratory, Immunology Frontier Research Center (IFReC), Osaka University, Yamadaoka 3-1, Suita, Osaka 565-0871 Japan; 2 Open and Transdisciplinary Research Institute (OTRI), Osaka University, Yamadaoka 3-1, Suita, Osaka 565-0871 Japan

**Keywords:** Microscopy, Raman spectroscopy, CD4-positive T cells, CD8-positive T cells

## Abstract

The monitoring of dynamic cellular behaviors remains a technical challenge for most established techniques used nowadays for single-cell analysis, as most of them are either destructive, or rely on labels that can affect the long-term functions of cells. We employ here label-free optical techniques to non-invasively monitor the changes that occur in murine naive T cells upon activation and subsequent differentiation into effector cells. Based on spontaneous Raman single-cell spectra, we develop statistical models that allow the detection of activation, and employ non-linear projection methods to delineate the changes occurring over a several day period spanning early differentiation. We show that these label-free results have very high correlation with known surface markers of activation and differentiation, while also providing spectral models that allow the identification of the underlying molecular species that are representative of the biological process under study.

## Introduction

Measurement techniques in biology are always subject to trade-offs between maximizing their specificity and spatio-temporal resolution, minimizing their invasiveness, while at the same time ensuring a sufficient throughput, both in terms of data rate and sample amount for statistical significance. In the context of single-cell data acquisition, the well-established technique of fluorescence-activated cell sorting (FACS) has provided numerous invaluable results thanks to its high specificity, based on fluorescent dyes and very high throughput^1^. Recent commercial instruments can reach several million cells per minute, multiplexed with up to a dozen fluorescent signals^2^, owing to the commercial availability of a wide range of functionalized dyes. The coupling with advanced fluidics has also enabled high-throughput physical separation of different cellular phenotypes, which is standardly used in current wet lab protocols.

This approach is, however, reaching its limits in terms of multiplexing, as the amount of dyes that can be measured is limited by the bandwidth available for detection, and the spectral overlap between emission widths of each fluorescent dye, which require increasingly complex compensation procedures. Solutions circumventing these issues have recently been proposed through various technical means, such as intensity-based fluorescent barcoding with advanced compensation procedures^3^, or the use of narrower emitters, including quantum dots^4^ or Raman tags^5^. Nevertheless, positive isolation—implying that the target population is stained—can cause multiple issues, especially in the context of clinical applications. Negative isolation is also possible, but due to the complexity of the isolation cocktails, it is limited to purification of highly standard cell populations, making this approach not suitable for targeted applications.

Another technique that is becoming highly prominent in the field is single-cell sequencing, which allows extremely high specificity through a wide range of measured parameters^6,7^. The technique however requires balancing the inner variability of the samples, in terms of individual expression, with the high cost per sample, which also limits the overall throughput. Contrarily to optical methods, these techniques are also destructive, which makes the study of dynamic behaviors challenging.

We show here how a purely optical label-free method, namely Raman micro-spectroscopy, can be used to study subtle cellular changes in a non-invasive way^8^, and specifically study the activation and differentiation of live T cells. This technique provides a highly multivariate signal that is very suitable for combination with machine learning methods for advanced analysis^9^. Since the signals are label-free, originating from endogenous contrast, it can be challenging to reach high specificity. Nevertheless, such approaches have recently been successfully used for high-throughput cell screening^10,11^, and applied to study the immune response of lymphocytes, either based on an imaging approach with a relatively low amount of samples^12^, or a method relying on averaging the signal within a single cell^13^, applied on T cell lines^14^. A comprehensive study of the in vitro effects of various inflammatory drugs on immune cells has recently been reported^15^. Raman spectroscopy has also been employed ex vivo to measure the response of T cells upon LPS-induced inflammation^16^.

We show here how label-free high-throughput single-cell Raman measurements can reach specificity high enough to be used to study in vitro T cell differentiation, and to decouple the effects of activation and differentiation. We first demonstrate how our measurements correlate with standard marker expression, where the measurements display clear temporal patterns. We then study the effects of T cell activation, to show how it can be separated from the influence of differentiation, where each process can be identified by different dynamics and response. Finally, we further validate our results by comparing our signals with the ones from effector cells differentiated in vivo, and develop models that match the long-term temporal behaviors of differentiation.

## Results

The results of this article were generated using a custom multimodal platform designed to acquire single-cell Raman spectra at high-throughput, parts of which were reported previously to assess the immune cellular responses of macrophage cells^11,17^. To ensure high repeatability during the measurements performed across multiple days, the system was fully automated to allow data acquisition across multiple fields of view without any manual intervention (see Supplementary Fig. S1 and “Materials and methods” for details), yielding an acquisition rate in the order of 1000 cells/h, mostly limited by the exposure time of Raman spectra acquisition.

**Figure 1:**
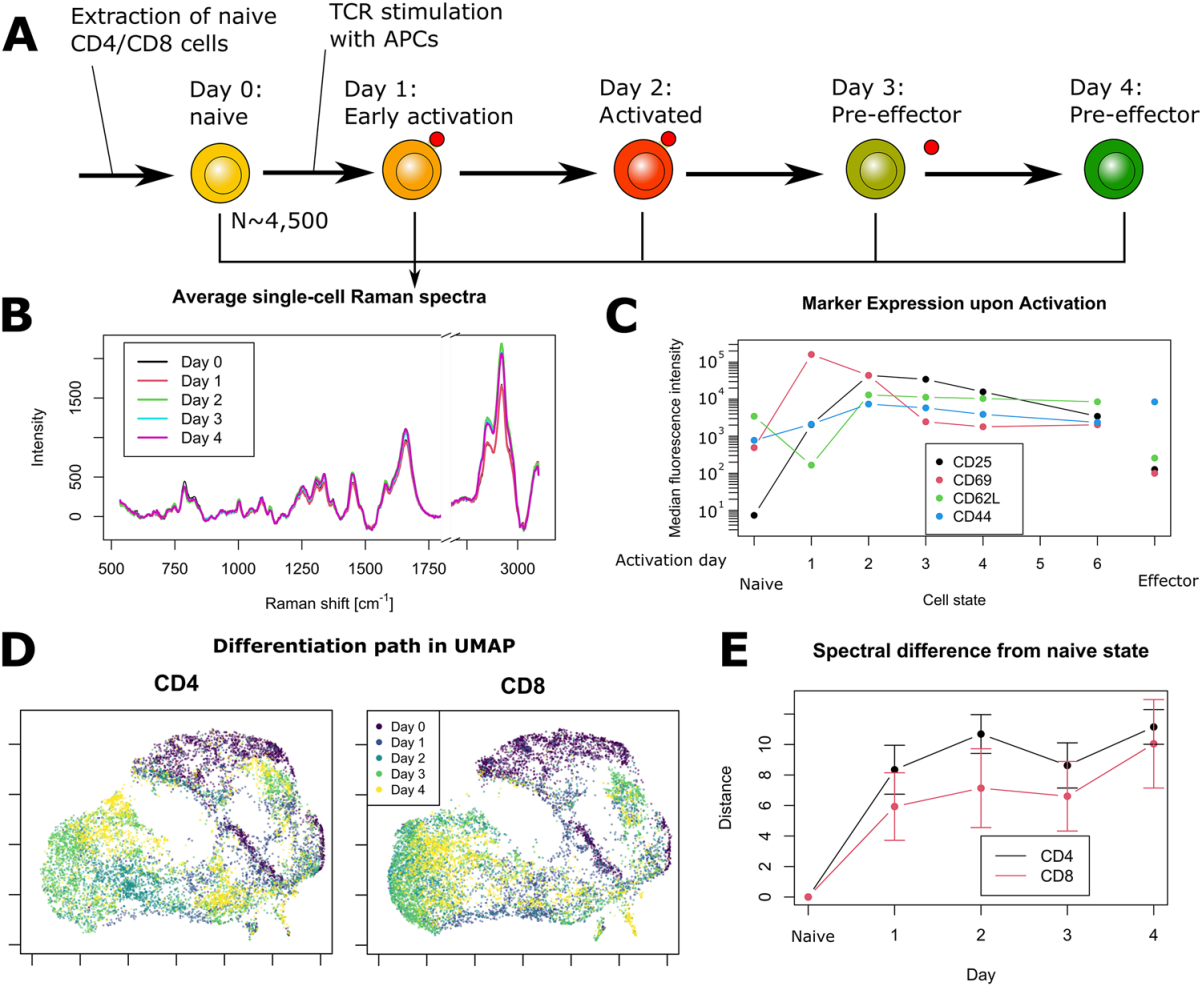
Early T cell differentiation monitored with Raman spectroscopy. (**A**) Experimental protocol, where murine T cells are stimulated with artificial APCs and measured everyday during 5 days. (**B**) Average single-cell Raman spectra (approximately 2000 cells per day and per type), showing that only small changes can be observed during the onset of activation. (**C**) Median fluorescence intensity signal derived from the expression of surface markers of CD4 cells over time, representative of T cell activation and differentiation. (**D**) UMAP decomposition of Raman data, where the gradual evolution of the signal upon activation can be readily observed. Differences between phenotypes (CD4/CD8) are also clearly visible. All results are representative of at least 3 experiments. (**E**) Quantification of the daily signal changes with the Mahalanobis-like distance. Curves are the average of 3 experiments, error bars indicate standard deviation.

To monitor the early response induced by T cell activation and subsequent differentiation, naive murine CD4 and CD8 cells are cultured with artificial antigen-presenting cells (aAPCs) that bind to the T cell receptor (TCR) and provide primary and co-stimulatory signals. Cells are then measured separately every day with a daily throughput of 2000–2500 cells per phenotype where they are activated and differentiate into pre-effector cells over several days, as illustrated in Fig. 1A. Furthermore, to validate the protocol, cells cultured in parallel and treated identically, are used to measure the expression of surface markers that represent T cell activation (CD25/CD69) and differentiation (CD62L/CD44). Cellular Raman spectra are endogenous signals that originate from molecules located within cells, while the numerical aperture and spectrometer slit effectively suppress out of focus signals, together implying that surface markers do not directly contribute to that signal. On the other hand, considering that cell processes usually involve a cascade of molecules, the ones occurring in the cytoplasm and nucleus can be considered as upstream, and measured in the Raman signals, while surfaces markers are downstream indicators of related processes.

As Raman spectra are often complex and, as stated above, generated by all intracellular molecules, we employ dimensionality reduction methods to analyze our measurements. We focus on unsupervised methods in order to study the inherent trends and separations between groups that are present in our data without introducing a priori information in the models. We employ essentially principal component analysis (PCA), a well-established method to decompose multivariate data based on its variance, and uniform manifold approximation and projection (UMAP), a more recent method based on non-linear dimension reduction that has displayed great performance to emphasize local structure in data^18^. We then also generate classification models based on regularized logistic regression, which we identified as a very reliable method for cellular Raman data^19^.

### Raman indicators can monitor early T cell differentiation

Simple inspection of the average Raman signal per day (see Fig. 1B) shows that spectral differences between days are relatively small, and are essentially smaller than the cell-to-cell variation. There are nevertheless some tendencies that can be observed, such as the increase in overall signal, especially in the C–H stretching region after day 2, which can be attributed to the augmentation of cell size that occurs upon activation. This increase is concomitant with the peak of CD25 expression, as shown in Fig. 1C, where the median fluorescence intensity (MFI) is displayed for different surface markers of CD4 cells, representative of cell activation and differentiation (the daily FACS plots from which the MFI are derived are displayed in Supplementary Fig. S2). The expression across the 5 days of stimulation is compared with naive cells (corresponding to day 0) and in vivo effector cells, which are characterized by CD62L^−^/CD44^+^ expression in the case of CD4 cells.

To analyze the evolution of the intracellular content monitored by Raman, we consider the UMAP plots derived from all the data measured across 5 days, and displayed separately for each cell type in Fig. 1D, where the gradual changes can be readily identified in an approximately circular clockwise pattern. Interestingly, both phenotypes follow a very similar path, where they rapidly separate from naive cells upon activation, and create various local clusters upon early differentiation by day 4, which is closely correlated with the overall CD25 expression, which peaks by day 2 before slowly decreasing.

While UMAP is a very powerful tool that allows the study of the local data structure, its non-linearity can make interpretation difficult. We therefore also study the global structure in the PCA space, and computed the Mahalanobis-like distance of each day cluster to the naive state (see Fig. 1E), which shows that, as in UMAP, activated cells rapidly separate from naive ones, with the distance increasing by day 4, instead of getting closer, showing that the circular pattern of UMAP is not representative of global tendencies. Again, it is interesting to note that both CD4 and CD8 cells follow the same overall temporal behavior.

### Activation yields significant changes in Raman spectra

**Figure 2:**
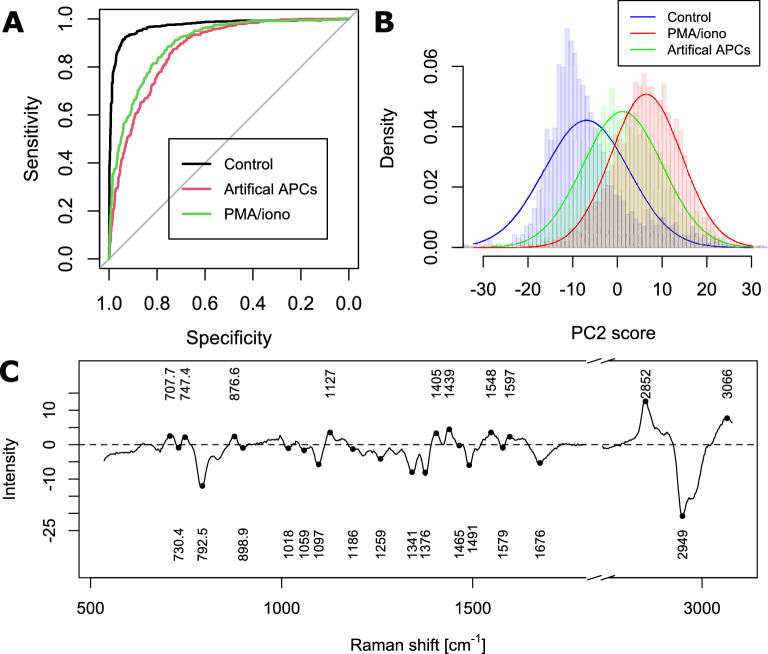
Detection of T cell activation. Cells activated through chemical means or TCR stimulation are compared to resting (unstimulated) cells. (N = 2500 per class for training) (**A**) ROC curve of classification performance. (**B**) Single-cell populations distribution along the PCA scores of PC2, which contains most of the variance related to activation. (**C**) Loading vector of PC2.

In order to separately study the effects of T cell activation and early differentiation, we measured naive CD4 cells stimulated during 24 h with either aAPCs, or a cocktail of PMA/ionomycin known to bypass the TCR stimulation to activate multiple intracellular signaling pathways that provides a very potent stimulation^20^. We validated the cellular response at a population level by measuring the IL-2 secretion culture medium (see Supplementary Fig. S3). We then generated a multinomial supervised model that can classify activation as well as the stimulation methods (2500 samples per class for training). As displayed in the receiver operating characteristic (ROC) curves of Fig. 2A, generated by testing the model with 20% of the data, activation can be detected with very high accuracy (92.4%), although it is much more challenging to identify the type of stimulation, with a discrimination between aAPC and PMA/ionomycin treated groups of only approximately 70% accuracy. While this demonstrates the sensitivity of Raman to detect cellular activation, it also shows that the measured changes are mostly intracellular, as the TCR stimulation induced by aAPCs generates relatively small changes in the spectrum compared to the purely intracellular signaling induced by PMA/ionomycin.

To further analyze the effect of activation, we consider the PCA decomposition of the data, where most of the variance induced by the effects of stimulation is located in PC2, as shown in Fig. 2B. Cells stimulated with PMA/ionomycin display higher scores than TCR stimulation, which is consistent with the external validation of IL-2 secretion which is also larger in that case, indicating a stronger activation at a population level (see Supplementary Fig. S3), which suggests that the derived markers are correlated with the intensity of the activation response. The main molecular changes involved in the case of activation are represented by the loading vector of this component, as shown in Fig. 2C. The vector mostly indicates a loss of signal from DNA/RNA with several peaks representative of nucleic acids (792, 1376 cm^−1^^21,22^) and backbone (1097 cm^−1^^23^), whereas the few positive peaks indicate an increase of amino-acids (877, 1127 cm^−1^^24,25^) and protein structure (1405 cm^−1^^26^) upon stimulation, which is in agreement with recent results reported for PMA stimulation^15^. It is also possible to identify strong contributions from CH_2_ stretching, either symmetrical (2852 cm^−1^) or asymmetrical (2949 cm^−1^)^27^. On the other hand, there is surprisingly only little agreement between these results and what has been previously reported^12,14^, perhaps due to the difference in cell type; DO11.10 TCR transgenic mice in Ref.^12^, Jurkat cell line in Ref.^14^, compared to C57BL/6N wild type mice used in our study. We also note a significantly larger sample size employed here.

### CD4 and CD8 cells follow a similar path towards differentiation, but are getting gradually separated

**Figure 3:**
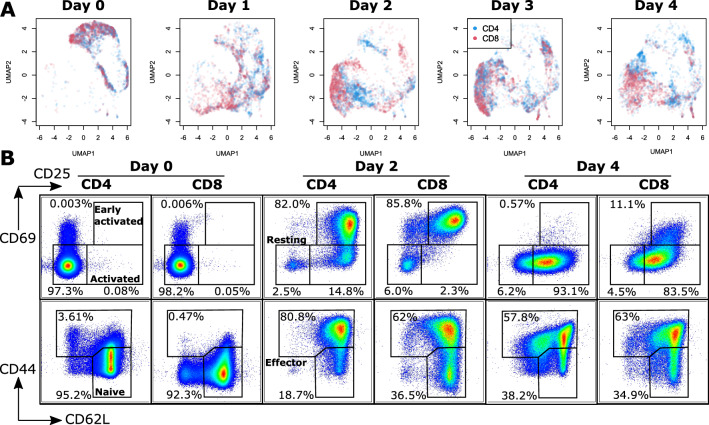
Cellular evolution over 5 days upon activation. (**A**) UMAP plots of T cells stimulated across several days, displayed separated by day. (**B**) Parallel measurements of the expression of surface markers for activation (CD25/CD69) and early differentiation (CD44/CD62L) separated per day, with cell types, and corresponding FACS panel showing activation/differentiation. The results are representative of at least three experiments.

The UMAP plots displayed in Fig. 1D showed that cells (both CD4 and CD8) follow a general trajectory upon activation, but the details are hard to visualize due to the large amount of data points in the plot. The results separated by days are displayed in Fig. 3A, where the large changes that occur can be readily identified. At day 0, cells are contained in a fairly localized region in the upper-right corner of the projection, and both phenotypes are mostly overlapped, without much difference between them. As shown in the corresponding FACS plots (see Fig. 3B), cells are resting (i.e. non-activated, characterized by CD25^−^/CD69^−^) and are in a naive state (characterized by CD62L^+^/CD44^−^), as expected by the purification procedure.

Upon activation, cells follow a downward trend (day 1), and reach the extreme lower-left corner of the projection plots by day 2, which corresponds to the peak of activation, with high expression of both CD25 and CD69 (see Fig. 1C). It is interesting to note that a small percentage of cells (< 10%) remains in a resting state, which is also visible in the UMAP plots as the small cluster located in the upper-right corner. Interestingly, resting CD4 and CD8 cells are in different locations at day 2, implying a difference in the spectral signature of activation for the two phenotypes. CD4 and CD8 cells are separated during the activation period, and CD8 cells appear to be following a faster path than CD4 ones, which would be consistent with reports that CD8 cells differentiate more quickly^28^. Each phenotype is also rather homogeneously distributed, which is consistent with the distribution in CD62L/CD44 expression plots.

After day 2, CD4 also reach the extreme lower-left of the UMAP plots (day 3), and by early differentiation (day 4), cells are starting to concentrate in small sub-clusters, and both phenotypes are completely separated. At this point, cells are not expressing CD69 anymore, which is representative of early activation. CD25 is also slightly decreasing, and sub-populations can be identified in differentiation markers, with most cells being CD62L^+^/CD44^+^, as in day 2, but another CD62L^mid^/CD44^+^ population starts to appear, in particular in CD4 cells. This is consistent with the emergence of a pre-effector population, which is characterized by high CD44 expression, and the identification of two main sub-populations, i.e. ‘central memory’ (CM) cells that are CD62L^+^, and ‘effector memory’ (EM) CD62L^−^ cells^29^, where CD4 effectors are mostly composed of EM cells, consistent with in vivo conditions, as discussed below.

To confirm that the observed changes are indeed due to biological effects, we also measured Jurkat cells over 5 days without stimulation, and performed UMAP decomposition, as previously. As shown in Supplementary Fig. S4, while some structure can be identified, the cell distribution over different days is mostly homogeneous, without any identifiable temporal pattern, showing that no external effect is inducing the trends observed for cellular activation.

The results are also highly reproducible, as shown by the plots of different experiments (see Supplementary Fig. S5), where independent decompositions lead to similar distributions, and where all the temporal trends discussed above can be identified too. Finally, we performed an experiment where the surface marker expression was measured every day to confirm the trends identified in Fig. 3B (see Supplementary Fig. S2). It is possible to see that most expression profiles are rather continuous as also illustrated by their MFI in Fig. 1C. We do observe that CD62L drops abruptly on day 1, however this is an effect known to occur upon early activation^30^. Levels on later days (day 6, see Supplementary Fig. S2) also show that measurements performed later are not necessarily relevant for biological interpretation, as activation levels further drop, which is a known effect of in vitro stimulation, where re-stimulation is often required. We could also observe that cell viability significantly decreased after day 5, especially in the case of CD8 cells.

To further study the changes occurring upon activation and early differentiation, we also quantitatively observed the score evolution of the first 12 components of the PCA decomposition (see Supplementary Fig. S6). It is possible to see that most scores have very clear temporal trends that match behaviors identified in the MFI of surface markers, again showing the correlation between the cellular response as measured by the endogenous Raman signal and the activation/differentiation state as measured by surface markers. Several scores for example reach an extreme value at day 2, the peak of activation, and maintain extreme levels (see PC1, 2, 3, 5, 6), while others reach extreme values at day 1 or 2, and return to basal levels afterwards (see PC4, 8, 11, 12). However, the complex behavior of each component, which is linked to a loading vector that contains multiple bands related to different molecular species (as in Fig. 2C), makes the derivation of quantitative tendencies challenging. These results also show the degree of reproducibility provided by our approach, as the displayed values are averages over several weekly experiments.

### Ex vivo effector cells are mostly closer to naive than in vitro stimulated cells

**Figure 4:**
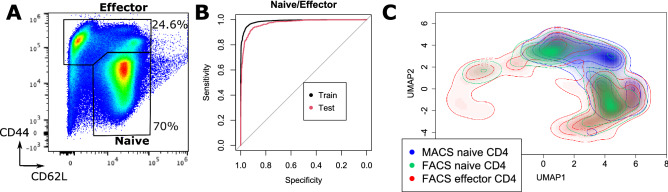
Comparison of cells differentiated in vitro and ex vivo effector cells. (**A**) CD62L/CD44 expression of CD4^+^ cell populations, with the gates employed for sorting naive and effector cells. (**B**) ROC curves of classification performance between naive and effector cells (N = 4000 for each class for training, test performed on 20% of the data). (**C**) Density plot of naive and effector cells retrieved by fluorescent cell sorting projected on the UMAP derived from cells displayed in Fig. 3A. Original naive CD4 cells retrieved by magnetic sorting (day 0, see Fig. 3A) are also displayed for comparison.

To further confirm how our previous results could be related to changes occurring in the case of cells differentiated in vivo, we separately retrieved naive and effector cells from CD4 populations by fluorescent sorting, according to the gates indicated in Fig. 4A. As fluorescent dyes can greatly disturb Raman signals, we first checked that the selected dyes had no influence on the cellular spectra. Raman measurement of control cells compared with cells stained with each individual dye did not show any significant difference and spectral differences were well below the standard deviation of cell-to-cell variations, with differences being within only a few percent of the whole signal (see Supplementary Fig. S7).

We first analyzed the Raman spectra to assess the level of differences that are present between naive and effector cells. While there was no substantial separation between the two populations in unsupervised decomposition such as PCA, it was possible to generate models that can accurately separate the two types with over 90% accuracy, as shown by the ROC curves (see Fig. 4B), demonstrating that there are substantial spectral differences between the two populations.

The spectral data from sorted cells was then projected onto the UMAP derived in Fig. 3A to compare the ex vivo cells with the ones previously stimulated in vitro (see Fig. 4C). As expected, naive cells extracted from both methods (negative MACS depletion and fluorescent sorting, respectively) are projected onto the same region. Similarly, effector cells appear to be mostly located in the same region as naive cells, which would indicate that the separation that is observed with in vitro stimulation is primarily due to activation, as ex vivo effector resting cells are not located in the same region (bottom-left). However, it is interesting to note that the cell distribution is very close to the CD4 cells differentiated at day 4 (see Fig. 3A), where the two largest clusters are located at identical places as effector cells, as shown in Supplementary Fig. S8, where the main sub-population are highlighted with their corresponding Raman spectra. Interestingly, differences such as the larger overall spectral intensities in the case of activated cells can be identified, as previously.

### Linear models allow the derivation of molecular indicators of differentiation

**Figure 5:**
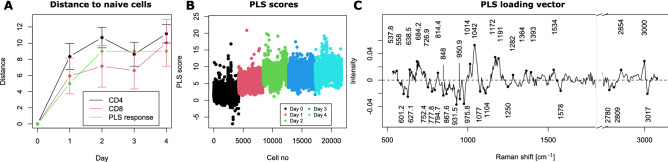
PLS model representing early differentiation. (**A**) Distance between cellular sub-populations, measured as a Mahalanobis-like distance and assigned matching response. (**B**) Single-cell scores derived from the PLS model, displaying the same increasing values as distances. (**C**) Resulting loading vector of the model, where peaks are representative of the activation and early differentiation.

While the analysis presented above shows the ability of single-cell Raman measurements to detect the small changes occurring upon T cell activation, it is not possible to derive molecular insights from the UMAP decomposition due to its high nonlinearity. Similarly, classification models as used in Fig. 2 or Fig. 4B are not suited to study non-binary cases which occur in gradual biological transitions. For this reason, we also use a partial least square (PLS) model where we employ a response that matches the distance that was previously measured between days (see Fig. 1E), where it gradually increases in the first 2 days before reaching a plateau, as displayed in Fig. 5A. This makes it possible to find a multivariate vector that provides scores matching the behavior occurring during activation, which can also be used for analysis.

The resulting model fits the defined response well, as shown in Fig. 5B, where the scores are in good agreement with the response, although some batch effects can be identified, in particular on day 2 and 4. This can also be explained by the fact that both CD4 and CD8 cells are included in the model, while it was previously identified that the two phenotypes were especially separated on those days (see Fig. 3A).

The resulting loading vector, which represents the linear response that creates scores matching the increasing distance between cellular populations upon activation, is shown in Fig. 5C. The vector is quite complex and contains a large amount of features, which is expected as it originates from a more advanced model. Nevertheless, the first striking point is that it very different from the PCA loading vector shown in Fig. 2C that represented activation, showing that other phenomena are involved. The interpretation is however quite difficult due to the complexity of the vector, which can make band assignments complicated, with the risk of over-interpreting some of the peaks observed. Furthermore, the projection is here performed on the whole data average, while it has been shown that multiple sub-populations are present, with distinct spectral profiles.

## Discussion

Optical spectroscopy has the ability to retrieve signals that are very rich in information, as illustrated by the results above, where changes occurring during cellular differentiation can be identified and used to monitor live cells. The power of such an approach becomes particularly visible when large numbers of cells are measured, which ensure high reliability of the statistical methods used to analyze the data and to perform predictions on new data sets. This can, however, be challenging to implement as it can be achieved realistically only with fully automated systems that are not commercially available at this stage.

There is a duality in how spectroscopic measurements can be used, being composed of a multitude of single bands that average together to create a complex and continuous signal representing the contribution of all molecules contained within the excitation volume. It can be exploited purely as a highly multivariate signal, to generate accurate models for classification, and to allow the identification of intricate behaviors with the use of nonlinear methods such as UMAP. Conversely, single bands can be used directly to interpret the molecular mechanisms of the phenomenon under investigation. While this aspect is used extensively in chemistry, where precise molecular changes can be studied, individual peak identification can be very challenging with spectra from biological samples, where many molecular species are co-located, superimposing their contributions. Nevertheless, even with challenging samples it is still possible to study specific band relevance by using the vectors resulting from multivariate analysis, as obtained here through PCA, logistic regression or PLS.

While the information content in the vibrational spectrum is very high, its specificity for functional studies can be limited. As illustrated by the UMAP plots in this article, small cellular changes can be readily analyzed, but the degree of separation is far less clear than what is classically obtained for example in the case of RNA-seq analysis, where phenotypes can typically be identified through standard cluster analysis^31^. However, one key advantage of optical methods is non-invasiveness, which allows for parallel analysis with complementary methods, which was not directly used in this study. It is possible for example to pair the label-free measurements at single-cell level with functional analyses based on immuno-fluorescence^17^.

The multivariate analysis and diverse ways of analyzing the data make it possible to first study relatively strong responses such as T cell activation, where very accurate detection models can be generated. Thanks to the clarity of the model, it is also possible to delineate the underlying molecular changes occurring upon activation, and identify a dose–response behavior depending on the strength of the stimulation.

More complex responses such as differentiation are more difficult to analyze, partly because of their longer dynamics. The use of non-linear algorithms coupled with larger data sets allows the identification of the day-to-day differences. While it is not possible to identify specific molecular species in that case, there are still clear specific changes with a dependence on the phenotype. There is also a very strong correlation between the label-free results and the expression of surface markers known to represent T cell activation and differentiation, demonstrating a strong consistency with expected known biological behaviors. This is especially interesting considering that, as stated above, the Raman signals themselves are not expected to be directly related to these surface markers, but rather are allowing identification of subtle cell states by way of other endogenous molecular changes.

In addition to these general trends, this approach is also specific enough to separate the effects of activation and differentiation, as shown by comparing the population distributions of stimulated cells with ex vivo effector cells, where clear differences between naive and effector cells can be identified with very accurate classification, and highly consistent results are obtained between ex vivo effector cells and in vitro pre-effector ones.

As demonstrated above, optical spectroscopy can be a very powerful approach for single live cell analysis, especially when coupled with automation to reach high numbers for statistical relevance and dynamic analysis. The measurement, rich in endogenous features, can be used in various ways as it provides both a highly multivariate signal for machine learning and quantitative variables for molecular interpretation. It is also specific enough to allow non-invasive identification of sub-clusters within homogeneous cellular populations.

Finally, we proposed at the end of our manuscript a method to attempt to reach molecular specificity in the case of highly complex behaviors, by employing PLS to model the surface marker expression and obtain a projection vector that can represent cellular differentiation. While the results obtained in this last part are highly preliminary, we believe that such approaches could further push the applicability of single-cell optical spectroscopy, by bridging the gap between the two usual fields of study that are usually employed in analytical chemistry, namely machine learning and molecular interpretation, to increase the specificity of these methods.

## Materials and methods

### Mice

C57BL/6N mice were purchased from Japan SLC and maintained under specific pathogen-free conditions. This study followed ARRIVE guidelines on the use of experimental animals. All animal experiments were conducted with the approval of the Animal Research Committee of the Research Institute for Microbial Diseases in Osaka University, Japan (approval no H29-02-0), and in accordance with the guidelines of the Animal Care and Use Committee of Osaka University. Female mice aged between 8 and 12 weeks were used for all experiments.

### Cell preparations

Single cell suspensions of splenocytes are obtained by filtering spleens from typically 2 mice through a 100 μm cell strainer with RPMI 1640 medium (Nacalai). Erythrocytes are eliminated through immersion in a RBC lysis buffer (Biolegend) on ice for 3 min, and naive CD4 and CD8 T cells are then purified with dedicated MACS kits (Miltenyi Biotec) used according to the manufacturer’s instruction with a manual separation column. Full CD4 populations (naive and effector) are retrieved with the same kit without employing CD44 microbeads.

For short (24 h) stimulations, T cells are suspended in a culture medium composed of RPMI 1640 supplemented with 10% fetal bovine serum (FBS, Gibco) and penicillin/streptomycin (Sigma-Aldrich) with 100 U/mL and 100 μg/mL, respectively. Cells are incubated in tissue-culture 96-well plates (Thermo Fisher, 100 μL per well) at a density of 10^6^ cells/mL, at 37°C in a humidified atmosphere containing 5% CO_2_. They are stimulated either with a cocktail of phorbol 12-myristate 13-acetate (PMA) and ionomycin (Sigma-Aldrich) at 10 ng/mL and 0.4 μM, respectively, or with CD3/CD28 activation beads (Gibco) prepared at a 1:1 density. Before further experiments (FACS or Raman measurements), beads are removed with a magnet (Thermo Fisher).

For long incubations (over 1 day), T cells are suspended in a culture medium prepared as above, but also supplemented with 55 μM of 2-mercaptoethanol (Sigma-Aldrich) and 6 ng/mL of recombinant murine IL-2 (PeproTech). Stimulation is performed with CD3/CD28 beads prepared at a 1:1 density, and the medium is refreshed and cells split to maintain viability when deemed necessary, typically every 2 days.

Jurkat cells (Riken BioResource Center) are cultured in RPMI 1640, and are plated on 10 cm tissue-culture dishes (Thermo Fisher) at a density of 1–2 × 10^5^ cells/mL and passaged every 2–3 days. All experiments were performed with cells from passage 10 to 13.

### Raman measurements

Cells are washed and resuspended at a density of 0.5–1 × 10^6^ cells/mL in phosphate buffered saline (PBS, Nacalai) supplemented with 2% FBS. They are then plated in 4-well micro-inserts (Ibidi) fixed on 35 mm quartz dishes (Matsunami). Raman measurements are performed with a system described previously^13,32^ that was modified to fully automate measurements.

Briefly, a 532 nm laser (Coherent) is used for excitation, by focusing it with a 60 × objective (Nikon, water immersion, 1.27 NA), yielding a power at the sample of 487.5 mW/μm^2^. The back-scattered light is collected by the objective, filtered with dichroic and notch filters (Semrock) before being injected into a 500 mm Czerny-Turner spectrometer (Andor) with a 300 lp/mm grating that spreads the spectral information onto a scientific CMOS Orca 4.0 detector (Hamamatsu), yielding a spectral resolution of approximately 10 cm^−1^. The spectrum of one single cell is acquired with an exposure time of 3 s.

Cells are simultaneously imaged with an off-axis digital holography system^33^ that delivers quantitative phase images (QPI) that are employed to selectively target cells in the field of view. They are excited with a Raman spot that is rapidly scanned within a region that covers approximately 60–90% of the cell body to retrieve a more representative single-cell spectrum, as previously described^13^.

The system is employing a motorized stage to automatically move between regions in a sequential manner (see Supplementary Fig. S1). In each field of view, cells are detected by segmentation of the QPI signal with a deep-learning based algorithm^34^ that ensures high reliability in detection of target cells for Raman measurements. The system is also periodically refocused by employing the digital propagation feature of QPI to compute a metric based on the variance of the amplitude image^35^ without requiring any sample movement during scanning.

### Antibodies, flow cytometry and cell sorting

All antibodies were employed according to manufacturers’ instructions. Anti-mouse PE conjugated CD4 (GK1.5), APC conjugated CD25 (PC61.5), Super Bright 600 conjugated CD62L (MEL-14), APC-e780 conjugated CD44 (IM7) were purchased from eBiosciences. Anti-mouse PE-Cy7 conjugated CD8 (53–6.7), FITC conjugated CD69 (H1.2F3), BV711 conjugated CD62L (MEL-14), BV421 conjugated CD44 (IM7) were purchased from Biolegend.

Cells are washed and resuspended at 10^6^ cells/mL in staining buffer (Thermo Fisher) and incubated with all surface markers for 1 h on ice. Viability is assessed by adding 0.5 μg/mL of 7-AAD (Biolegend) at least 10 min before measurements. Cells are analyzed on an Attune NxT Cytometer (Thermo Fisher), and compensation is performed with OneComp beads (Thermo Fisher) stained with single dyes. Sorted cells are purified on a Sony SH800S. All data is analyzed with FlowJo v10 (TreeStar).

### Data analysis

The original processing of Raman data is performed with scripts developed in the laboratory for Matlab (Mathworks). Spectra are first baseline corrected with cubic spline interpolation, and cosmic rays are removed through median filtering. The spectral range is then calibrated by interpolating spectra on a common grid based on a spectrum of pure ethanol measured each day. The silent region (1800–2700 cm^−1^) is then removed. If necessary, outliers (mostly composed of empty spectra) are manually removed by identifying them with PCA.

All processing is then performed with the *R* program^36^. Principal component analysis is done with built-in functions. Receiver operating characteristic (ROC) calculations, logistic regression, regularized with Lasso, uniform manifold approximation and projection (UMAP) and PLS are performed with the *pROC*^37^, *glmnet*^38^, *uwot* and *mixOmics*^39^ packages, respectively. UMAP is performed with a cosine distance metric, and applied on the first 25 principal components of the data. Other calculations are based on scripts developed internally.

Implementation details for Raman-based classification were given previously^19^. Briefly, processed Raman spectra are used directly to train the models of regularized logistic regression, with the regularization parameter being selected with tenfold cross-validation to minimize the amount of variables while keeping the binomial deviance within one standard deviation compared to the average minimum. Test data is always independent data that was not seen by the model during training, and multinomial models are generated in a One-vs-Rest way.

The distance between sub-populations (clusters) is computed as a Mahalanobis-like distance defined as

1 $${d}^{2}\equiv {\left({\overline{X} }_{i}-{\overline{X} }_{j}\right)}^{T}{\left({S}_{i}+{S}_{j}\right)}^{-1}\left({\overline{X} }_{i}-{\overline{X} }_{j}\right),$$

where ${\overline{X} }_{i}$ is the mean value of the population *i*, and ${S}_{i}$ is its empirical covariance matrix^40^.

## Supplementary Information

Supplementary Figures.

## Data Availability

All data needed to evaluate the conclusions in the paper are present in the paper and/or the Supplementary Materials. The raw data and datasets acquired and generated during the current study are available from the corresponding author on reasonable request.
