# Supplementary material for: Non-invasive monitoring of T cell differentiation through Raman spectroscopy
Source: Sci Rep. 2023 Feb 22;13:3129. doi: 10.1038/s41598-023-29259-8 (PMC9947172; doi:10.1038/s41598-023-29259-8)
Supplement: Supplementary file 1 — Supplementary Figures. [file 41598_2023_29259_MOESM1_ESM.docx]

Supplementary information for

Non-invasive monitoring of T cell differentiation through Raman spectroscopy

Nicolas Pavillon,^1,*^, Nicholas. I. Smith^1,2,*^

Biophotonics Laboratory, Immunology Frontier Research Center (IFReC),

Open and Transdisciplinary Research Institute (OTRI),

Osaka University, Yamadaoka 3-1, Suita, 565-0871, Suita, Osaka, Japan

[*n-pavillon@ifrec.osaka-u.ac.jp](mailto:*n-pavillon@ifrec.osaka-u.ac.jp), *[nsmith@ap.eng.osaka-u.ac.jp](mailto:nsmith@ap.eng.osaka-u.ac.jp)

**This file includes:**

Figs. S1 to S8


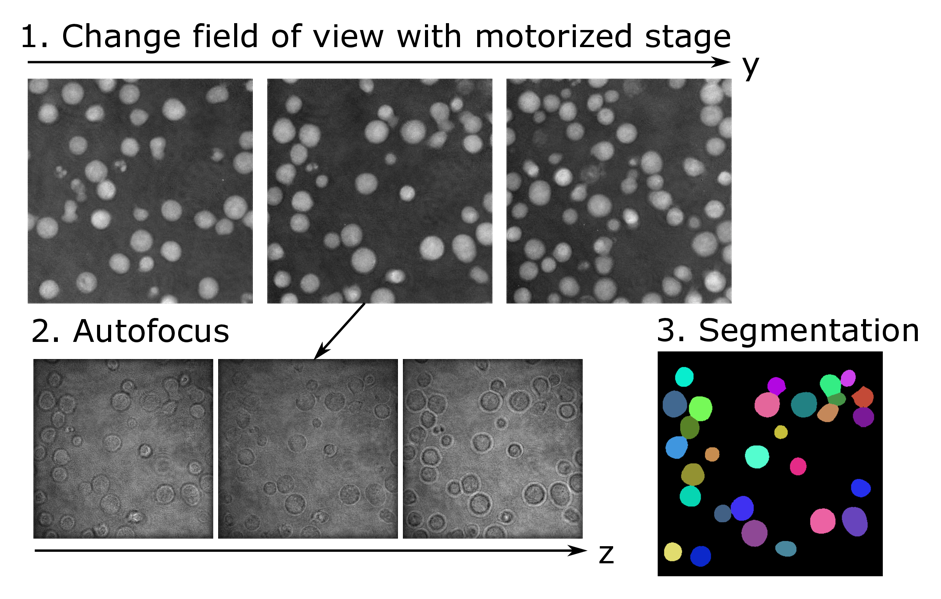


**Fig.** **S1.**

Schematic of system automation. The field of view is changed with a motorized stage, and imaged by quantitative phase. The focus is adjusted by minimizing variance in the amplitude image, and cells are then identified by segmentation. Their coordinates are then used to measure each cell with the Raman system.


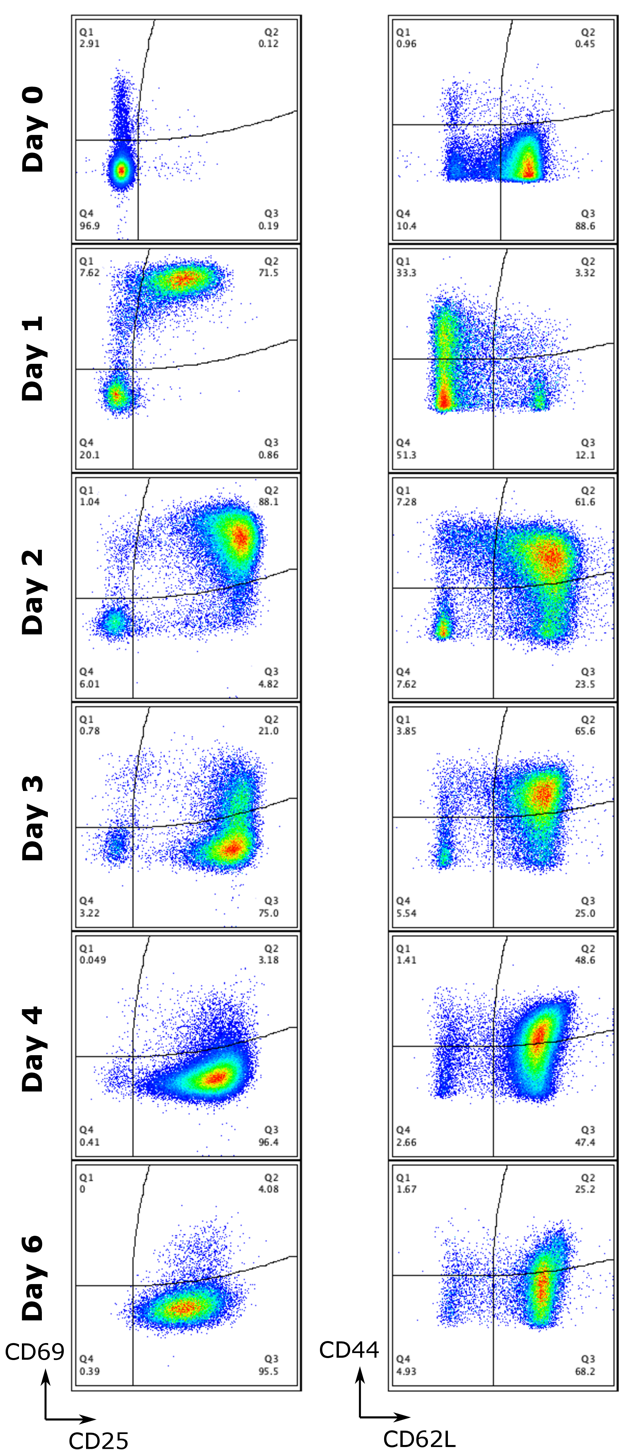


**Fig****. S2.**

Surface marker expression of CD4 cells measured everyday after stimulation by aAPCs.


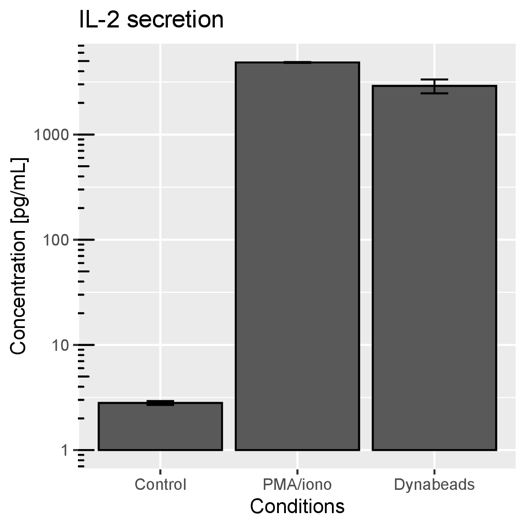


**Fig.** **S3.**

Secretion of IL-2 by CD4 cells (10^5^ cells/well) after 24h stimulation, measured by ELISA. Values are averages of at least three experiments, with error bars denoting standard deviation.


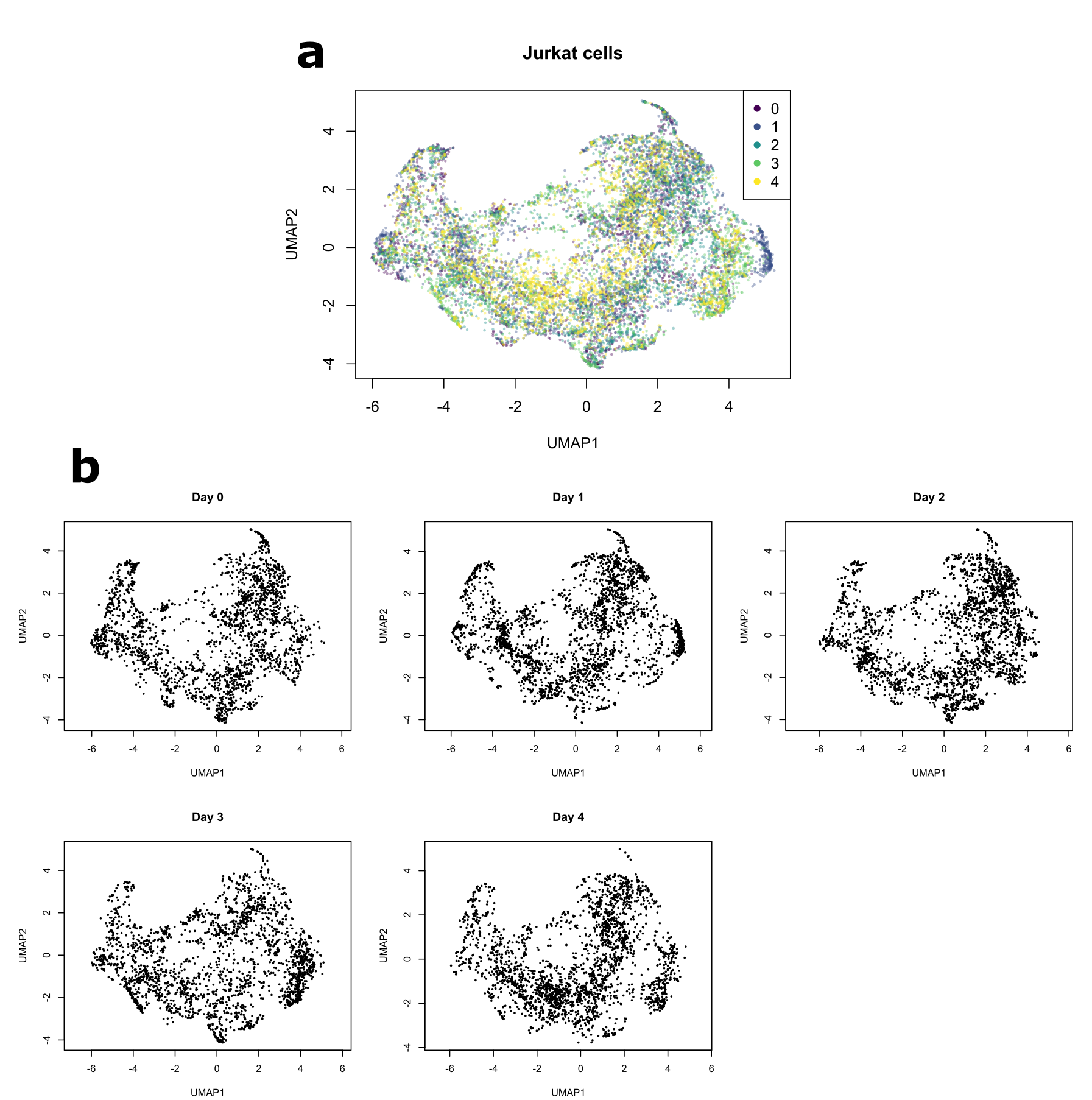


**Fig. S4.**

UMAP plot of Jurkat cells measured over 5 days. (**a**) All days displayed in one plot; (**b**) Days displayed separately.


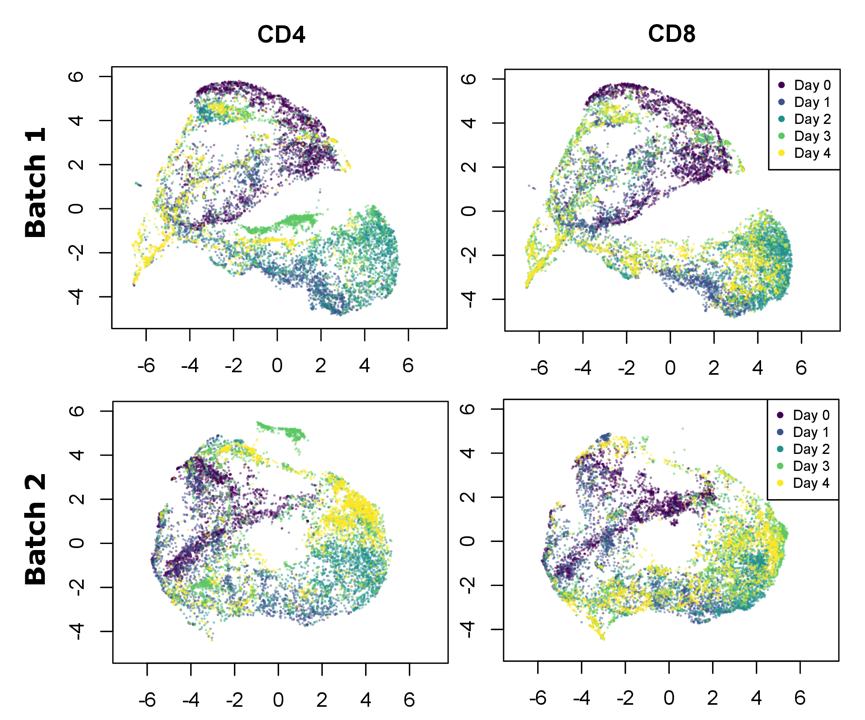


**Fig.** **S5.**

UMAP plot of additional experiments, with independent decomposition.


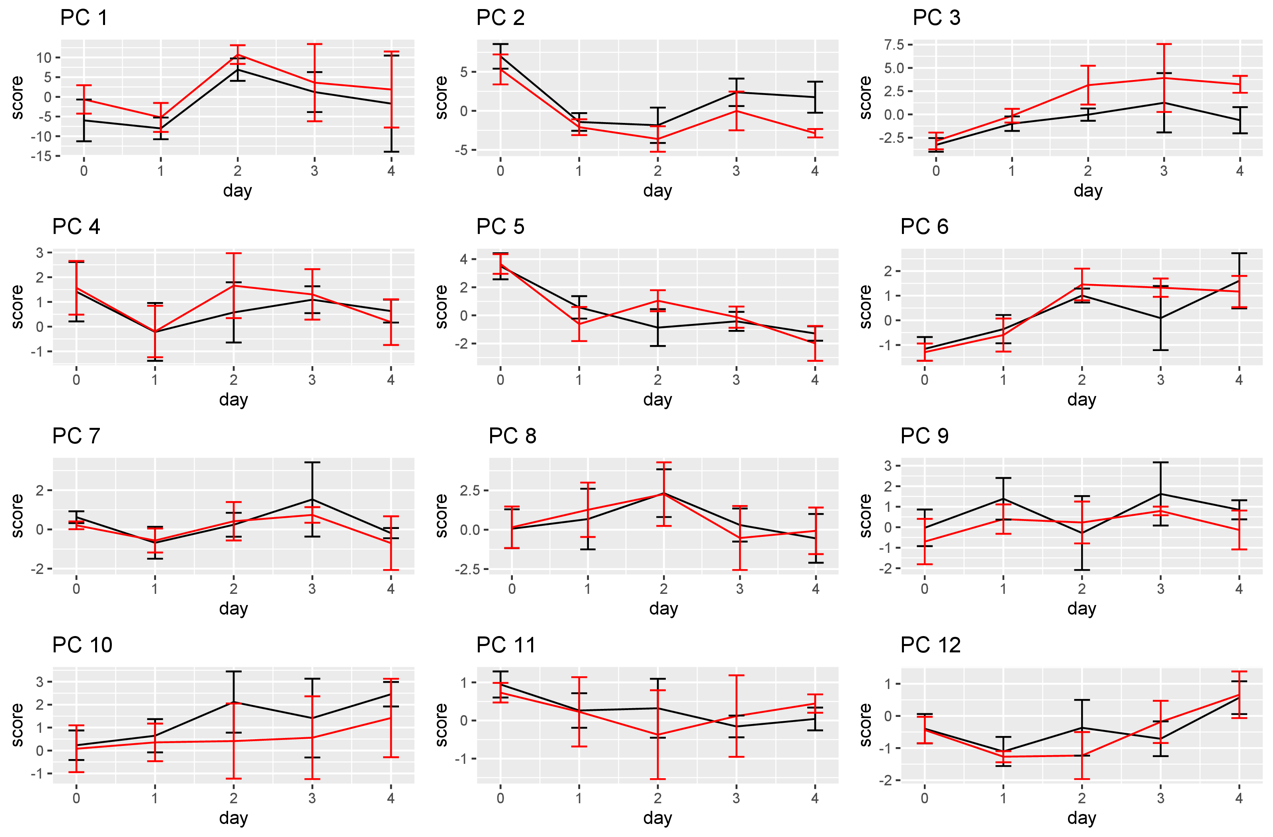


**F****ig. S6.**

Temporal trends of PCA scores for T cells activated over several days (black: CD4, red: CD8). Values are population averages for 3 independent experiments, error bars are day to day standard deviations.


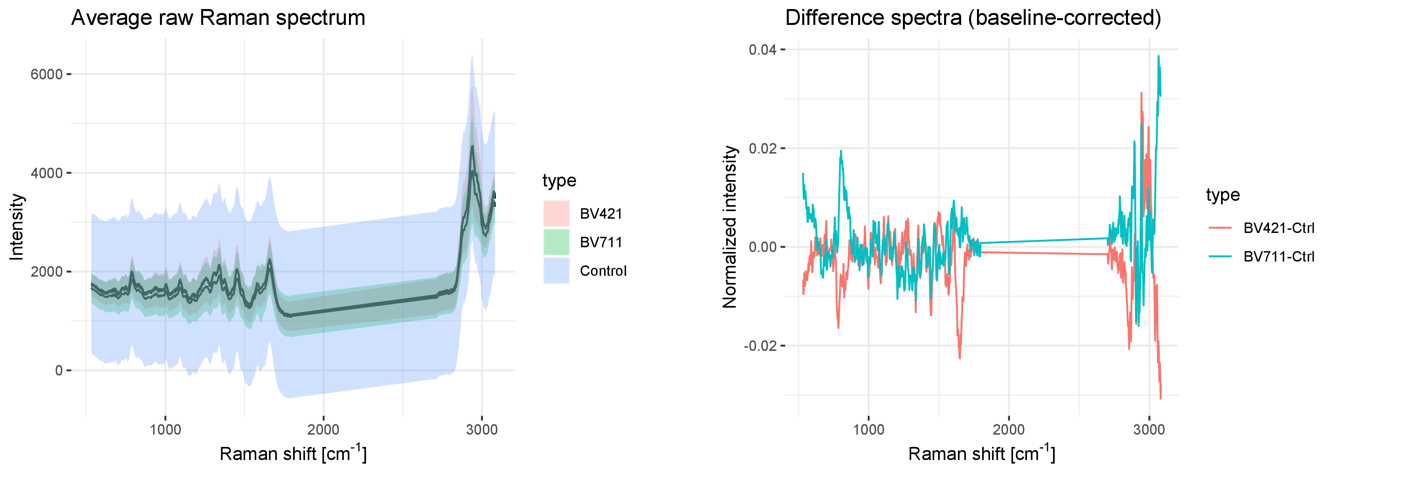


**Fig.** **S7.**

Control experiment showing no significant influence of fluorescent dyes on the Raman signal measurements. (left) Average raw Raman spectra of control and stained cells, shaded regions indicate standard deviation (N=1,200 for each class). (right) Difference of average spectra after baseline correction, between each stained cell populations and control.

**Fig****. S8.**

(**a**) Main clusters in CD4 cell distribution on the last measurement day shown in Fig. 3, with (**b**) their corresponding average Raman spectra.
